# Antisense Oligonucleotide-Mediated Reduction of HDAC6 Does Not Reduce Tau Pathology in P301S Tau Transgenic Mice

**DOI:** 10.3389/fneur.2021.624051

**Published:** 2021-06-28

**Authors:** Antonio Valencia, Veronica L. Reinhart Bieber, Bekim Bajrami, Galina Marsh, Stefan Hamann, Ru Wei, Karen Ling, Frank Rigo, H. Moore Arnold, Olga Golonzhka, Heike Hering

**Affiliations:** ^1^Biogen, Cambridge, MA, United States; ^2^Ionis Pharmaceuticals, Carlsbad, CA, United States

**Keywords:** HDAC6, KIGS, KXGS, tau pathology, tau acetylation, tau phosphorylation, ASO

## Abstract

Acetylation of tau protein is dysregulated in Alzheimer's Disease (AD). It has been proposed that acetylation of specific sites in the KXGS motif of tau can regulate phosphorylation of nearby residues and reduce the propensity of tau to aggregate. Histone deacetylase 6 (HDAC6) is a cytoplasmic enzyme involved in deacetylation of multiple targets, including tau, and it has been suggested that inhibition of HDAC6 would increase tau acetylation at the KXGS motifs and thus may present a viable therapeutic approach to treat AD. To directly test the contribution of HDAC6 to tau pathology, we intracerebroventricularly injected an antisense oligonucleotide (ASO) directed against HDAC6 mRNA into brains of P301S tau mice (PS19 model), which resulted in a 70% knockdown of HDAC6 protein in the brain. Despite a robust decrease in levels of HDAC6, no increase in tau acetylation was observed. Additionally, no change of tau phosphorylation or tau aggregation was detected upon the knockdown of HDAC6. We conclude that HDAC6 does not impact tau pathology in PS19 mice.

## Introduction

Abnormal post-translational modifications have been associated with the cytosolic accumulation and a gain of toxic function of the microtubule-associated protein tau in Alzheimer's Disease (AD) and other tauopathies (1). Numerous studies have investigated the association of different post-translational modifications with tau toxicity [reviewed in (2, 3)] and multiple acetylation sites on tau have been described and suggested to influence tau accumulation and aggregation (4–8). A recent publication by Arakhamia et al. compared cryo-EM structures and posttranslational modifications present on tau filaments purified from brains of patients with AD (9) and corticobasal degeneration (CBD) (10) showing that acetylation was found mostly within the fibril-forming core and was predicted to favor β-strand stacking and make tau less soluble (4).

Even though acetylation levels of tau appear to be dysregulated in AD, the functional significance of the disease-associated changes in this modification is controversial. For instance, an acetylation mimicking mutation on K174 slowed down tau turnover and caused memory deficits in mice (7). Similarly, tau acetylation mimicking mutations on K274 and K281 promoted memory deficits and impaired hippocampal long-term potentiation (LTP) (8). Additionally, tau fragments, upon acetylation by CREB-binding protein (CBP), or a K174Q acetylation mimetic promoted fibril formation *in vitro* (5, 7). On the other hand, acetylation of tau by histone acetyltransferase p300 reduced tau filament formation (11). The same authors identified K259 and K353 as acetylation sites on tau that are modulated by the opposing actions of p300 and histone deacetylase (HDAC) 6 (11). Acetylation at K259 and K353 was suggested to attenuate tau aggregation by regulating tau phosphorylation at the nearby sites S262 and S356 (11). In a follow-on study, ac-K321 was identified as another site for HDAC6-mediated deacetylation and was shown to be essential for inhibiting tau aggregation *in vitro* and preventing phosphorylation of downstream serine 324 (12). These studies suggested that increasing the acetylation of tau on specific lysine residues via the inhibition of HDAC6 may provide therapeutic benefit with regards to toxicity associated with tau phosphorylation and aggregation. In support of this hypothesis, overexpression of HDAC6 enhanced tau accumulation and a decrease in HDAC6 activity promoted tau clearance (13).

HDAC6 is a cytoplasmic HDAC with several identified substrates, including tau (14). HDAC6 interacts with tau *in vitro* and in human brain tissue and HDAC6 protein levels have been reported to be increased in AD cortex and hippocampus (14). Multiple small molecule HDAC6 inhibitors have been described in the literature and have shown efficacy in preclinical models of AD. Inhibition of HDAC6 reduced tau phosphorylation in various cell models (12, 14, 15) and reduced levels of aggregated tau in hAPP695/d and hTauP301L-transfected SH-SY5Y cells (15). *In vivo*, the HDAC6 inhibitor Tubastatin A reduced tau pathology and decreased total tau levels in rTg4510 mice (16). Tubastatin A and ACY-1215, another HDAC6 inhibitor (17), were efficacious at reducing levels of tau phosphorylation and A?, and improving spatial learning and memory in APP/PS1 mice (18). The selective HDAC6 inhibitor ACY-738 was also tested in APP/PS1 mice and led to a decrease in tau phosphorylation and amyloid pathology as well as improved axonal transport and memory (19). MPT0G211, a recently described novel selective inhibitor of HDAC6 reduced tau phosphorylation and aggregation, and ameliorated learning and memory deficits in 3xTg mice (20).

Although HDAC inhibitors with selectivity for paralog 6 have been identified (21–25) they may not be devoid of off-target activity and may suffer from pharmacokinetic properties potentially unsuitable for demonstrating CNS efficacy, and, when used at high doses, may lose their selectivity toward other members of the HDAC family. The use of antisense oligonucleotides (ASO) for gene knockdown (KD) provides a great and rapid alternative to traditional gene knockout and pharmacological approaches. ASOs against CNS targets, when delivered intracerebroventricularly in mice, show wide distribution throughout the brain and typically result in significant reduction in the expression of the target protein (26, 27). In this study we have utilized ASO technology to reduce levels of HDAC6 in the brains of PS19 tau transgenic mice to study the effects of HDAC6 knockdown on tau acetylation, phosphorylation and aggregation. We demonstrate that despite highly effective KD of HDAC6 in the brain, the levels of acetylated tau remained unaltered and tau pathology unaffected, suggesting that altered HDAC6 activity in the brain does not play a role in tau pathogenesis.

## Materials and Methods

### Human Brain Tissue

Human brain tissues from 10 male and female non-demented subjects (Braak Stages 0–III; referred to as “Control”) and 10 male and female AD (Braak Stages V–VI) subjects were obtained from the Netherlands Brain Bank. The average age of the non-demented subjects was 81.8 years and that of AD subjects was 77.9 years. For Western blot analysis tissue sections from the superior frontal gyrus were used. Post-mortem delay ranged from 4.5 to 13 h. All tissue samples were obtained using consent forms that approved the use of Human Biological Samples for research purposes.

### Animals

Tau transgenic mice with the P301S tau mutation (PS19) were purchased from Jackson Laboratory (Bar Harbor, ME, USA; strain B6;C3-Tg(Prnp-MAPT^*^P301S)PS19Vle/J (28). Wild type animals from the same colony were used. HDAC6 knockout animals were generated using a CRISPR/Cas-mediated genome engineering approach as described in (29, 30) and utilizing guide RNAs targeting exon 14 of the HDAC6 gene. Mice were maintained in a 12 h light/dark cycle at 67–74°C and 70% relative humidity, and standard diet of irradiated Purina 5P76 (from Pharmaserv) was administered *ad libitum*. All care and use of animals were in accordance with a protocol approved by the Biogen IACUC and adhered to all applicable guidelines and regulations (Cambridge Ordinance 1086, PHS Policy, AWA/AWAR, and the Guide).

### Oligonucleotide Synthesis

Synthesis and purification of all chemically modified oligonucleotides were performed as previously described (31). The MOE [2′-O-(2-Methoxyethyl)] gapmer ASOs are 18 (or 20) nucleotides in length, wherein the central gap segment comprising eight (or ten) 2′-deoxyribonucleotides is flanked on the 5′ and 3′ wings by five 2′ MOE modified nucleotides. Internucleotide linkages are phosphorothioate interspersed with phosphodiester, and all cytosine residues are 5′-methylcytosines. The sequence of the ASOs are as follows: Control-ASO, 5′- CCTATAGGACTATCCAGGAA−3′; HDAC6-ASO, 5- GCCTACTCTTTCGCTGTC-3′.

### ASO Delivery by Intracerebroventricular Injection

Mice were anesthetized with isoflurane (5% in air mixture), placed in a stereotaxic apparatus and prepared for surgery by shaving the animals' heads from the occipital crest forward to the eyes and out to the ears. The surgical area was cleaned with iodine. Each injection was 10 μl total volume per mouse at 2 μl/min flow rate and delivered 300 μg of control or HDAC6 ASO. The first injection was done at the age of 6.1 months, the second injection at 7.6 months, and animals were euthanized at 9.1 months of age. Stereotaxic coordinates of the injection were: −0.3 mm anterior and 1 mm lateral to bregma, depth of 3 mm.

### Euthanasia and Tissue Collection

The latest IACUC Guidance for the Humane Euthanasia of Laboratory Animals guidelines were followed, in addition to the IACUC Guidance for Guillotine Use and Maintenance. Animals were euthanized by live decapitation to prevent tau phosphorylation due to hypothermia induced by other euthanasia methods, including chemical cocktails and gas exposure (32–34). Promptly after decapitation, brains were dissected on ice and divided into two halves. The left hemi-brain was weighed and frozen on dry ice and used for biochemical analysis; the olfactory bulb and cerebellum were discarded. The right hemi-brain was transferred into a container of 10% formalin and used for histopathology analysis. With the progression of tau pathology, animals present motor deficits, showing paresis and eventually paralysis. To prevent the suffering of mice, those animals which showed severe paresis that impeded their ability to reach food were euthanized and excluded from the study.

### Real-Time Reverse-Transcription Polymerase Chain Reaction

Total RNA was isolated from mouse tissues and real-time reverse-transcription polymerase chain reaction (qRT-PCR) was performed as previously described (35). Approximately 10 ng RNA was added to EXPRESS One-Step SuperScript qRT-PCR Kit (ThermoFisher, Waltham, MA) with Taqman primer and probe sets: *Hdac6* forward primer: GGCTGGTCTATGATGAGAAGATG; *Hdac6* reverse primer: GACACATGATGCGTAAGATGC; *HDAC6* probe: TGGGACAATCATCACCCTGAGACAC; *Gapdh* forward primer: GGCAAATTCAACGGCACAGT; *Gapdh* reverse primer: GGGTCTCGCTCCTGGAAGAT; *Gapdh* probe: AAGGCCGAGAATGGGAAGCTTGTCATC. The *Hdac6* expression was normalized to the housekeeping gene *Gapdh* and this was further normalized to the level in vehicle treated mice.

### Protein Extraction

Human brain tissue from the frontal cortex from individuals with AD and controls was sonicated twice for 20 s in 10 × w/v RIPA buffer plus protease and phosphatase inhibitors (Roche, Basel, Switzerland), in a cold room. Homogenates were centrifuged for 10,000 rpm at 4°C for 10 min and the supernatant was collected for further analysis. Mouse hemi-brains were sonicated twice for 10 s each in a cold room in 10 × w/v buffer consisting of 10 mM Tris/HCl pH 7.5, 0.8 mM NaCl, 1 mM NaF, 1 mM Na3VO4, complete Protease Inhibitor Cocktail and PhosSTOP (Roche, Basel, Switzerland), and 10 μM anacardiac acid (Sigma-Aldrich, St. Louis, MO, USA). This homogenate was termed crude homogenate (CH). CH was divided for Western Blot and Mass Spectrometry analysis.

### Sarkosyl Fractionation

CH fractions were centrifuged at 1,500 rpm for 10 min at 4°C to discard debris. Samples were centrifuged at 10,000 rpm and the supernatant (SN) was used to measure soluble proteins and tau aggregation. The pellet was washed in Tris buffer and with 10% sucrose and sonicated as in described in section **Protein Extraction** in the cold room. 1% sarkosyl was added to the samples and incubated for 1 h at 37°C and was called the total sarkosyl fraction (TS); 200 μl aliquots were collected and stored at −80°C. The rest of the TS was centrifuged in a L90K Ultracentrifuge (Beckman Coulter, Brea, CA, USA) at 100,000 x g in a fixed angle rotor (Beckman 50Ti) for 1 h at 4°C; this supernatant was termed the sarkosyl soluble fraction (SS). The pellet was air-dried and resuspended in 80 μl of Tris buffer with protease and phosphatase inhibitors and termed the sarkosyl insoluble fraction (SI). Protein levels from each fraction were measured using the Pierce BCA Protein Assay Kit (Thermo Fisher Scientific, Waltham, MA, USA).

### Western Blot

Electrophoresis was done using equipment, reagents and protocols from Life Technologies (Woburn, MA, USA). Samples were prepared with 4 × loading buffer containing reducing agent and incubated for 10 min at 70°C. Ten mg of total protein was loaded per lane unless otherwise indicated. Electrophoresis was performed in 4–12% BisTris gels with MES running buffer (Life Technologies) supplemented with antioxidants, depending on the gel size, at a constant 200 V for 22 min for mini-gels or 45 min for midi-gels. Proteins were transferred onto the PVDF membranes using iBlot2 (Thermo Fisher Scientific) at a constant 20 V for 7 min. Membranes were incubated in Odyssey? Blocking Buffer (Li-COR Biosciences, Lincoln, NE) for 1 h at room temperature. Membranes were incubated in primary antibodies diluted in blocking solution plus 0.2% Tween 20 overnight at 4°C. Membranes were washed twice for 30 min each in TBS plus 0.2% Tween 20 (TBST). Membranes were incubated with the secondary antibodies for 1 h at RT followed by TBST washes as above. Membranes were washed once in dH_2_O for 5 min and imaged using Odyssey Cx-Li-COR system (Li-COR Biosciences). Antibody details and dilutions can be found in Supplementary Table 1. The polyclonal anti-acetylated tau KIGS antibody was kindly provided by Dr. Len Petrucelli at the Mayo Clinic (Jacksonville, FL, USA).

### Tau Aggregation HTRF Assay

The HTRF analysis was performed using the tau aggregation assay kit from Cisbio (Bedford, MA, USA). Protein was extracted from PS19 mice brain samples as previously described. 10 μl of sample containing 5 μg of total protein were added to a 384-well white low-volume plate (Corning Inc., Corning, NY, USA). Kit-provided anti-tau-d2 conjugate and anti-tau-terbium cryptate conjugate were diluted 1:50 in diluent buffer. Five μl each of anti-tau-d2 conjugate and anti-tau-terbium cryptate conjugate were added to each well. The plate was spun briefly and incubated at room temperature for 2 and 24 h. The plate was read on an Envision plate reader (PerkinElmer Inc., Waltham, MA, USA) and analyzed by plotting the ratio of 665 nm/620 nM × 10,000.

### Tau Enrichment, Digestion, and LC-MS Analysis

The CH fraction was centrifuged at 10,000 rpm for 10 min, supernatant and pellet were collected for tau immunoprecipitation (36). The pellet was solubilized in PBS containing 0.2% SDS and 0.1% NP-40 and further diluted with PBS to final 0.1% SDS and 0.05% NP-40 concentrations. Tau was enriched from both supernatant and re-solubilized pellet (referred to as “pellet” hereafter), using an in-house developed human tau-specific antibody ch6C5 (37). The antibody was crosslinked to agarose resin (Thermo Fisher Scientific) using provided reagents and instructions with minor modifications. Briefly, 500 μL of supernatant or pellet was mixed with 40 μL of ch6C5-conjugated resin (1:1 slurry) and incubated at 4°C overnight. The enriched tau was eluted from supernatant samples with 50 μL of 8 M urea, or with 50 μL of NuPage LDS Buffer containing 2-Mercaptoethanol and 50% elution buffer from pellet samples.

Tau-enriched eluants were divided into two halves, for trypsin or AspN digestion, respectively. The supernatant-originated eluates were reduced (10 mM dithioerythritol, 3 h), alkylated (22 mM Iodoacetamide, 1 h, dark), and diluted in 50 mM NH_4_HCO_3_, 150 mM NaCl, 5% Acetonitrile, before digestion with trypsin or AspN, at protease-to-protein ratio of 1:25 (w/w) for 15 h. Enzymatic digestion was stopped in 0.5% TFA. The pellet-derived eluates were first run on 4–12% Bis Tris SDS-PAGE gel for cleanup. Following gel staining, tau-containing bands were cut out, diced, and de-stained with 50/50 Acetonitrile/50 mM NH_4_HCO_3_ solution. Subsequently, reduction, alkylation and trypsin/AspN digestion were performed as described above but in an in-gel format. Peptides in the gel were extracted with 40/60 Acetonitrile/0.1% formic acid and dried in speed-vac. Both in-solution (for supernatants) and in-gel (for pellets) digested peptides were further cleaned (desalted) using C18 StageTips, speed-vac dried and stored at −20°C.

Prior to LC-MS analysis the dry peptide mixtures were reconstituted in 2% acetonitrile/0.2% formic acid solution and analyzed on a LC-MS/MS platform composed of a nanoLC system coupled Q Exactive HF (QE HF) mass spectrometer (ThermoFisher). Peptides were separated on an C18-AQ column (75 μm × 50 cm, Reprosil-Pur C18-AQ, 1.9 μm) and analyzed by QE HF at parallel reaction monitoring (PRM) mode for 11 selected peptides (Supplementary Table 2). The LC-MS/PRM data was processed using open-source software Skyline (MacCoss lab, Univ. of Washington), and chromatographic peak areas were output and used as relative measures of tau acetylation and phosphorylation at specified sites for detecting changes between HDAC6 ASO and control-treated samples.

### Immunohistochemistry

Brain hemispheres were cut sagittally, fixed in formalin, and embedded in paraffin. For each sample, 5 μm sections (50 μm apart each) were selected for IHC analysis. These sections were prepared and placed on charged slides, after which they were de-paraffinized and rehydrated. Heat-mediated epitope retrieval was performed in Ventana CC1 buffer (pH 8.0). Endogenous peroxidase activity was blocked by peroxide and endogenous biotin activity was blocked by Biotin Blocking Solution (Roche Ventana). Sections were incubated with hu40E8, a human IgG1 antibody which is specific for tau phosphorylated at S202/T205 (37), at a final concentration of 0.07 μg/mL, followed by incubation with secondary biotinylated goat anti-human IgG (Vector Labs). Staining was detected with 3,3′-diaminobenzidine (DAB) and sections were counterstained with hematoxylin.

Stained slides were digitized using the Pannoramic 250 Scanner, and images were analyzed with Visiopharm software. Relative area values for each animal were determined by calculating the geomean of five sections. Unpaired *t*-tests were performed using GraphPad Prism software.

### *In situ* Hybridization

*In-situ* hybridization was performed on 5 μm formalin-fixed, paraffin-embedded sections, using the RNAscope 2.5 LS Assay (Advanced Cell Diagnostics) on the Leica Bond RX autostainer. The probe for murine *Hdac6* was purchased from ACDBio (#422878). Detection of staining was done using RNAScope 2.5 LS Reagent Kit-RED, and sections were counterstained using hematoxylin to visualize nuclei.

### Cortactin Immunoprecipitation

Acetylated (and total) cortactin in mouse brain, as detected by the anti-acetyl cortactin antibody (Millipore 09881) in Western blots shown in **Figure 3D** and Supplementary Figure 5A, appeared to have a significantly smaller molecular size (~50 kDa) than the reported size of ~80 kDa. Smaller isoforms of the cortactin protein expressed in brain have been reported (38). To confirm the specificity of the antibody and that the ~50 kDa band corresponds to cortactin, hemibrains from HDAC6 wild type [WT (*n* = 2)], heterozygous [HET (*n* = 3)], and hemizygous [HEMI (*n* = 2)] mice were processed to generate crude homogenate as described in Section Protein Extraction and immunoprecipitated (using the Thermo Fisher Crosslink IP Kit) with two different antibodies against cortactin, either anti-cortactin antibody 05–180 (Millipore; Billerica, MA, USA) or anti-cortactin antibody sc-55579 (Santa Cruz Biotechnology; Dallas, TX, USA). The samples were eluted using kit-provided elution buffer and combined 1:1 with 25 μl of NuPage LDS Buffer (Life Technologies) containing 2-Mercaptoethanol (Sigma-Aldrich). Western Blots were run using 15 μl of the eluate, as described in Section Western Blot, and probed with either Millipore anti-cortactin antibody (05–180; Supplementary Figures 5B,D) or Millipore anti-acetyl-cortactin antibody (09881, Supplementary Figures 5C,E) at a dilution of 1:1,000. The Millipore anti-cortactin and anti-acetyl cortactin antibodies recognized the same ~50 kDa band in all IP samples confirming that the ~50 kDa is indeed cortactin.

## Results

### Acetylation of Tau Is Decreased and Phosphorylation Is Increased at the KXGS Motifs in Brains of Subjects With Alzheimer's Disease

Tau can be acetylated at multiple sites in mice and in humans (39, 40), but the functional significance of this post-translational modification for disease pathogenesis is not understood. An inverse relationship of tau acetylation and phosphorylation has been reported for the KXGS motifs within the repeat-domains of tau (11), which have been implicated in microtubule stabilization (41, 42) as well as tau aggregation (9) and it was suggested that increasing tau acetylation could be a mechanism for protection from tau aggregation (11).

**Figure 1:**
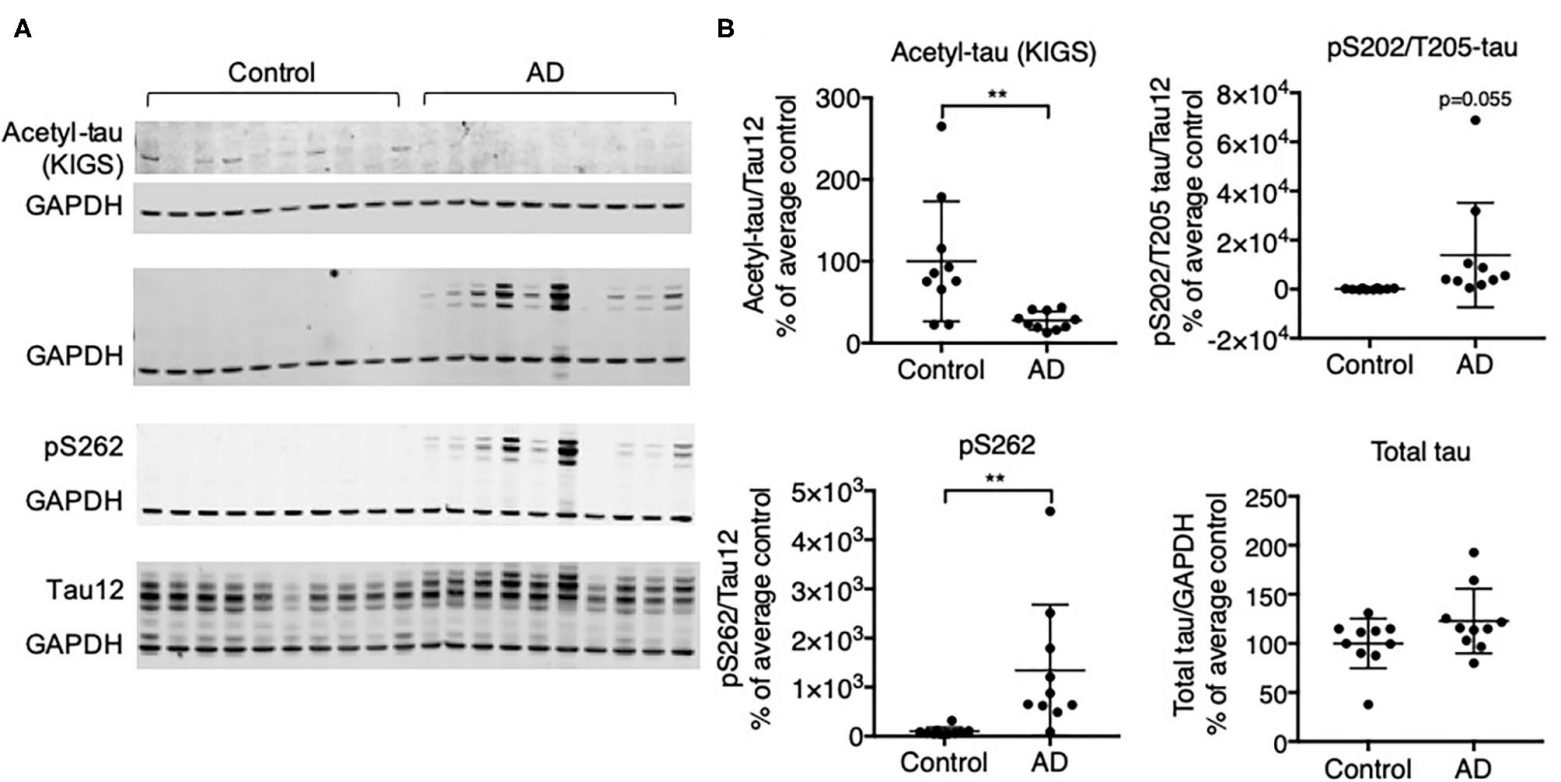
Protein expression of tau acetylated at KIGS inversely correlates with phosphorylated tau in brains from subjects with AD. **(A)** Representative Western Blots and **(B)** Western Blot quantification of acetyl-tau (KIGS), pS202/T205-tau (tau phosphorylated on Serine 202 and Threonine 205), pS262 tau, and total tau in control and AD patient brains (*n* = 10). Data is presented as mean ± SD and was analyzed using an unpaired non-parametric *t*-test. **p < 0.01.

We investigated the relationship of acetylation and phosphorylation at the KXGS motifs in human brain tissue from healthy subjects and subjects with AD utilizing a previously described antibody designed to specifically recognize acetylated lysine residues K259 and K353 within tau's KIGS motifs [anti-ac-KIGS, a gift from L. Petrucelli, (11)]. We confirmed by Western blotting that tau is hyperphosphorylated at the pathology-associated phosphorylation sites S202/T205 [epitope recognized by AT8 antibody (43)] and S262, which are localized within the first KIGS motif, in AD brains (*n* = 10) as compared to control brains (*n* = 10, Figure 1, Supplementary Figure 1). In contrast, while acetylation of tau at the KIGS sites was detected in several control subjects, it was nearly undetectable in AD subjects (Figure 1, Supplementary Figure 1). While no inverse correlation of acetylation and phosphorylation could be detected at the individual subject level (data not shown), the decrease in acetylation and increase in phosphorylation in the AD group vs. the Control group is consistent with the previously reported inverse correlation between acetylation and phosphorylation of tau at the KIGS sites (11).

### Knockdown of HDAC6 Increases the Acetylation of Alpha-Tubulin and Cortactin

Recent data suggest that the acetylation of tau at the KXGS motifs is regulated by HDAC6 and impacts its phosphorylation and aggregation *in vitro* (11). To test if HDAC6 modulated tau acetylation and tau aggregation *in vivo* we knocked down HDAC6 expression using an ASO in adult wild type mice and mice that express the human mutant P301S form of tau (PS19) (28) and analyzed brains using biochemical and histological techniques.

**Figure 2:**
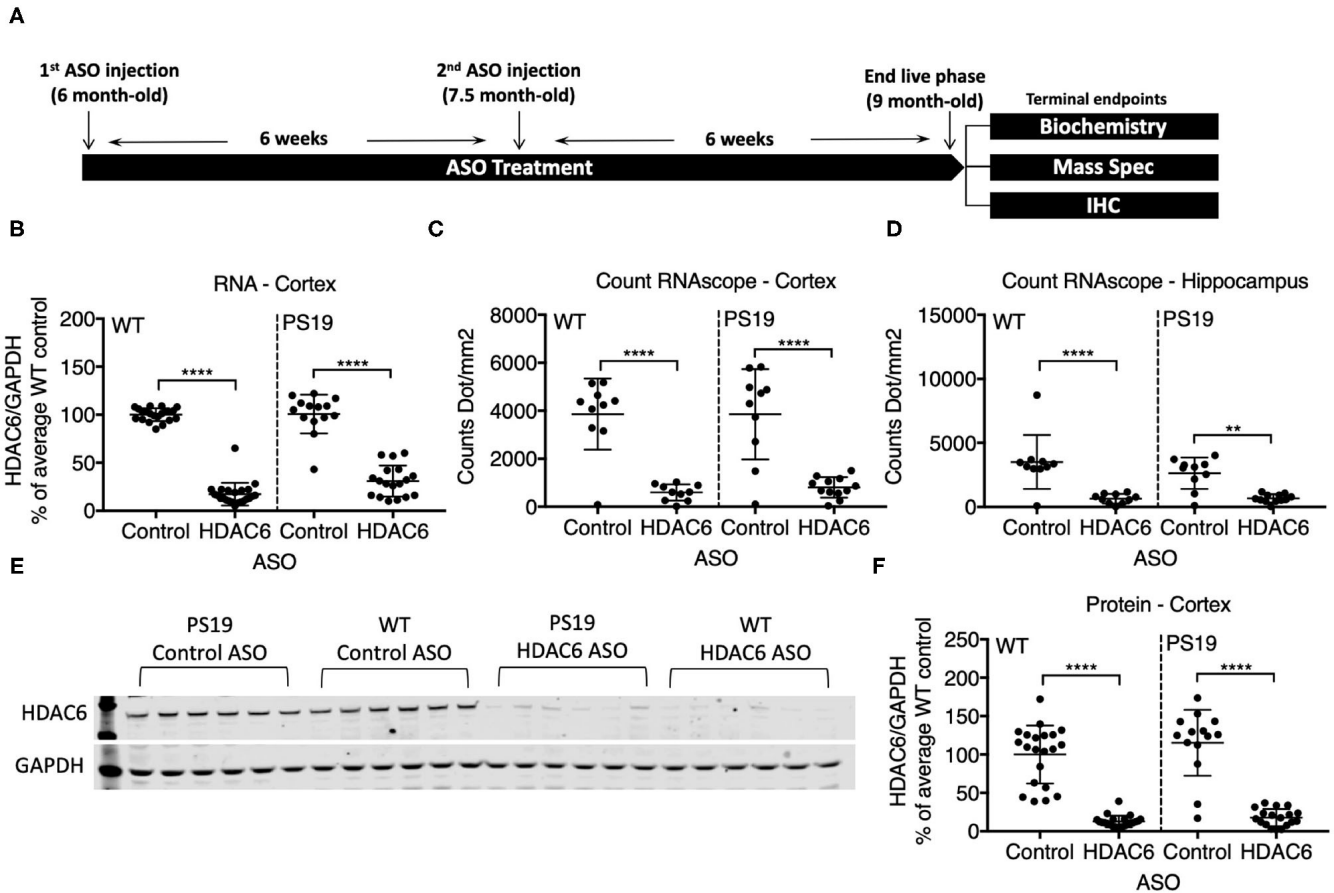
ASO treatment effectively knocked down HDAC6 RNA and protein levels in WT and PS19 mice. **(A)** Schematic of study design. **(B–D)** Quantification of HDAC6 expression in WT and PS19 mouse brains measured by qRT-PCR **(B)**, Count RNAscope in cortex **(C)**, and in hippocampus **(D)**. **(E)** Representative Western Blot and **(F)** Western Blot quantification of protein expression in brains of WT and PS19 mice treated with Control or HDAC6 ASO. Data is presented as mean ± SD and was analyzed using a one-way ANOVA with multiple comparisons *post-hoc* analysis. *n* = 21 and 23 for Control ASO- and HDAC6 ASO-treated WT mice, respectively, and *n* = 14 and *n* = 18 for Control ASO- and HDAC6 ASO-treated PS19 mice, respectively. **p < 0.01, ****p < 0.0001.

We identified RNase H ASOs that efficiently reduce the expression of HDAC6 mRNA in cultured Hepa1-6 cells (data not shown) and tested the most potent ones for target reduction in the CNS of mice. The ASO that was selected was confirmed to not reduce levels of HDACs 1, 2 or 3 (data not shown). PS19 mice and wild type littermates at 6 months of age were injected intracerebroventricularly with 300 μg of the selected ASO specific for murine HDAC6 or a non-targeting control ASO, followed by a second injection 6 weeks later (Figure 2A). 6 weeks after the second injection (at 9 months of age), animals were assessed for the extent of HDAC6 KD in the brain. This treatment paradigm was chosen because in 6-month-old animals only mild pathology is present, but by 9 months significant accumulation of tau pathology is observed. qPCR analysis revealed a strong reduction in HDAC6 mRNA levels to approximately 17% and 30% of control ASO treated animals in wild type and PS19 mice, respectively, after 12 weeks of ASO treatment in the cortex (Figure 2B; *p* < 0.0001 for WT and PS19). *In situ* hybridization with an HDAC6 specific probe confirmed the KD of HDAC6 mRNA in the cortex (Figure 2C; *p* < 0.0001 for WT and PS19; high magnification images in supplemental data, Supplementary Figure 8) and hippocampus (Figure 2D; WT: *p* < 0.0001; PS19: *p* = 0.011) in HDAC6 ASO treated mice. Similarly, HDAC6 protein levels were strongly reduced in HDAC6 ASO treated wild type and PS19 mice compared to control ASO treated mice (Figures 2E,F; *p* < 0.0001 for WT and PS19, Supplementary Figure 2), which correlated closely with HDAC6 mRNA reduction (data not shown). No difference in HDAC6 mRNA and protein levels was observed between control ASO treated wild type mice vs. control ASO treated PS19 mice (Figure 2B: *p* = 0.9985; Figure 2C: *p* > 0.9999; Figure 2D: *p* = 0.3034; Figure 2F: 0.3039), suggesting no increase in HDAC6 levels in mice harboring tau pathology.

**Figure 3:**
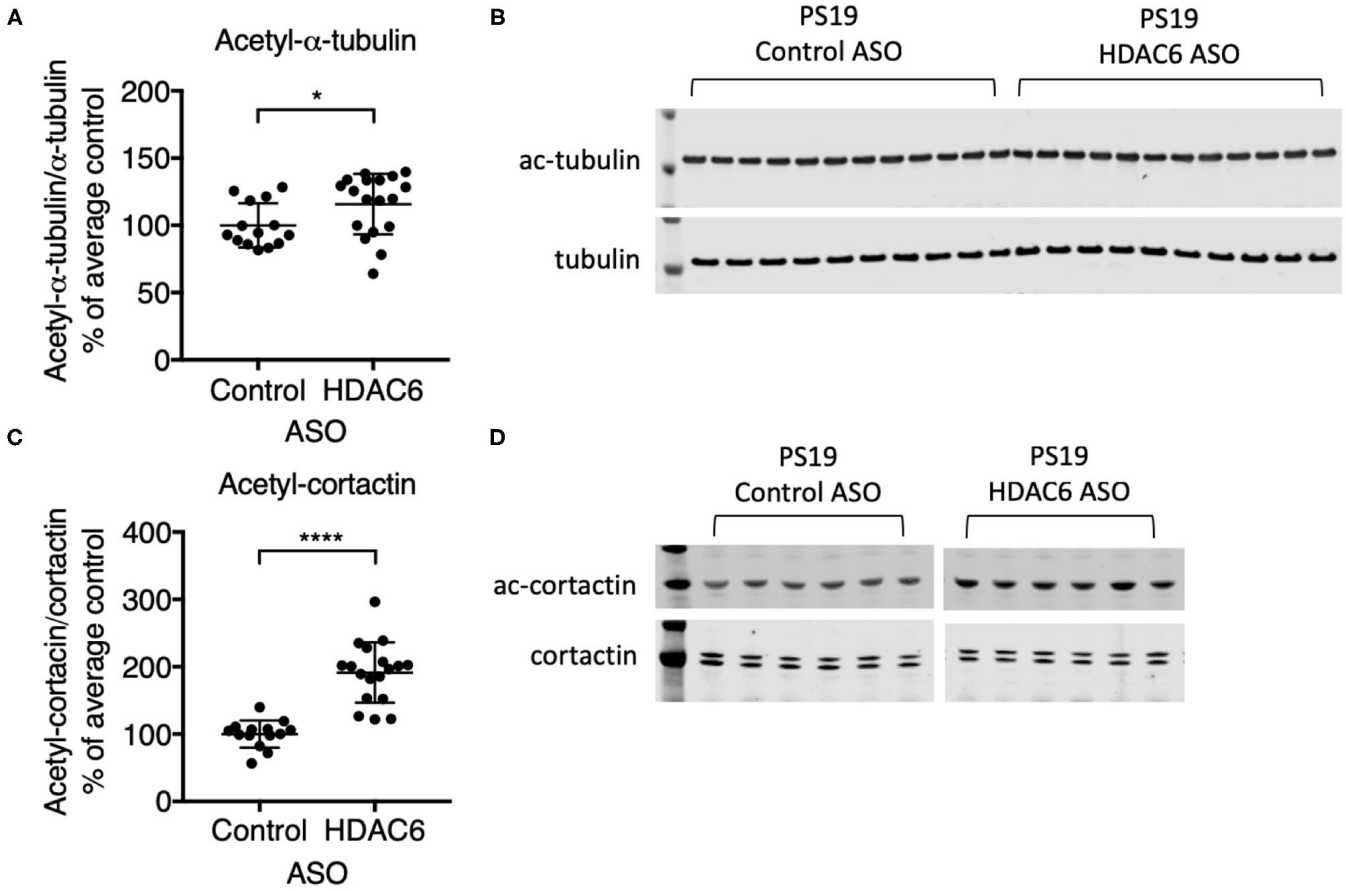
HDAC6 ASO treatment resulted in significant increases in acetylation of HDAC6 substrates. **(A,C)** Western Blot quantification and **(B,D)** representative Western Blots of acetyl-α-tubulin and acetyl-cortactin in brain samples from Control ASO-treated (*n* = 21) and HDAC6 ASO-treated (*n* = 23) WT mice or Control ASO-treated (*n* = 14) and HDAC6 ASO-treated (*n* = 18) PS19 mice. Data is presented as mean ± SD and was analyzed using an unpaired non-parametric *t*-test. *p < 0.05, ****p < 0.0001.

To demonstrate that ASO-mediated HDAC6 KD results in reduced substrate deacetylation, we assessed the acetylation levels of two known substrates of HDAC6. Alpha-tubulin is a well-described substrate of HDAC6 (24). HDAC6 knockdown resulted in a small, but significant increase in alpha-tubulin acetylation in the brains of PS19 mice (Figures 3A,B; *p* = 0.0363, Supplementary Figure 3). Acetylation levels of alpha-tubulin in the brain are high compared to other organs and thus HDAC6 KD may only be able to further increase acetylation levels of alpha-tubulin to a small extent. In contrast, HDAC6 knockdown elicited a robust increase in acetyl-cortactin, another known substrate of HDAC6 (44). Acetyl-cortactin levels were increased by ~1.9 fold in HDAC6 ASO treated PS19 mice compared to control ASO treated animals (Figures 3C,D; *p* < 0.0001; validation of the anti-acetyl-cortactin antibody using HDAC6 KO mouse brains is shown in Supplementary Figure 5). Together, these data suggest that knocking down HDAC6 for 12 weeks in brains of PS19 mice can cause sustained pharmacodynamic changes in substrates of HDAC6.

### Knockdown of HDAC6 Does Not Increase Tau Acetylation and Does Not Alter Tau Phosphorylation

**Figure 4:**
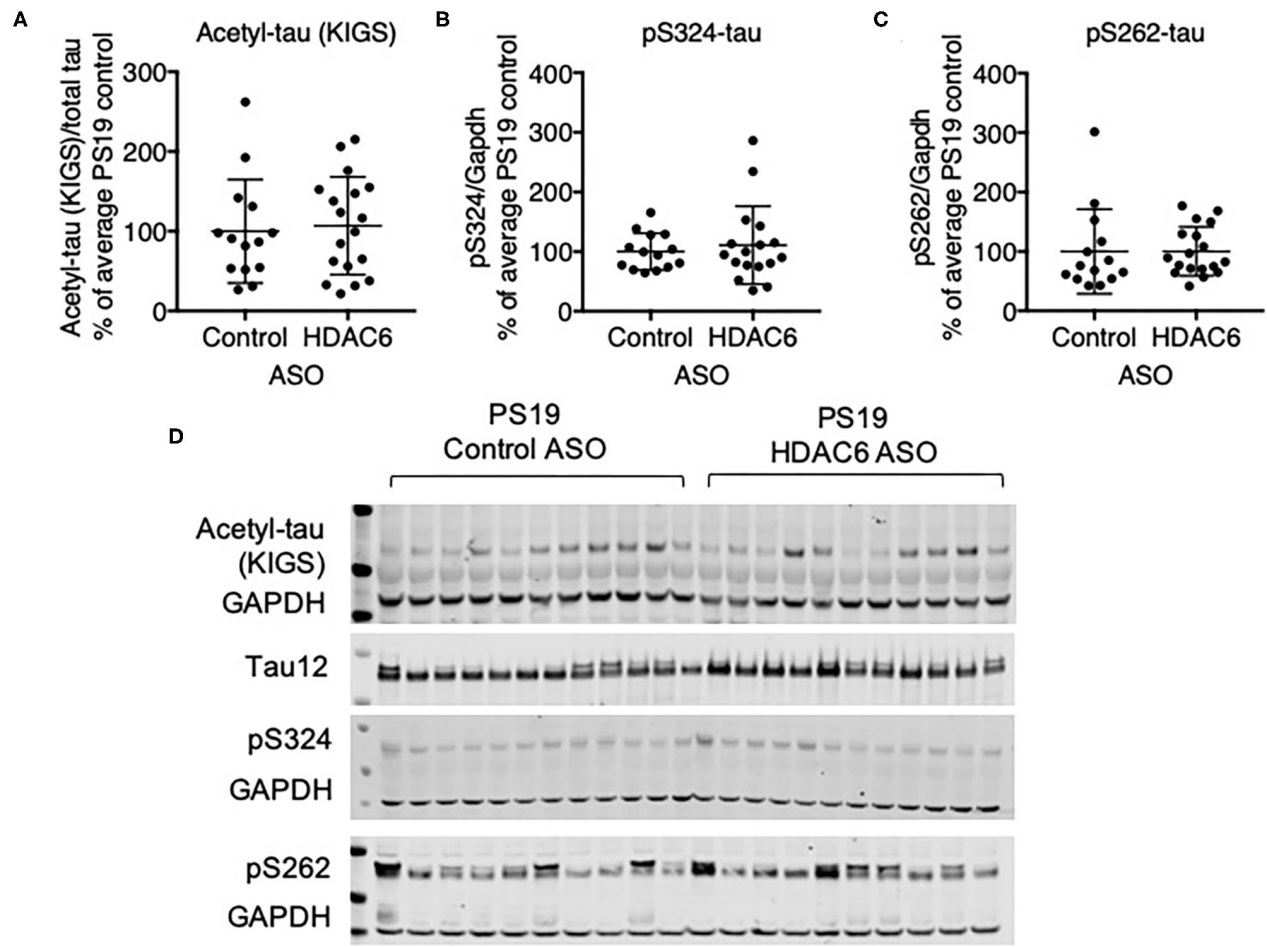
HDAC6 knockdown did not alter phosphorylation of tau in PS19 mice. **(A–C)** Western Blot quantification of pS262 tau, pS324 tau and acetyl-tau at KIGS, and **(D)** representative Western Blots from brains of PS19 mice treated with Control ASO (*n* = 14) or HDAC6 ASO (*n* = 18). Data is presented as mean ± SD and was analyzed using an unpaired non-parametric *t*-test.

**Figure 5:**
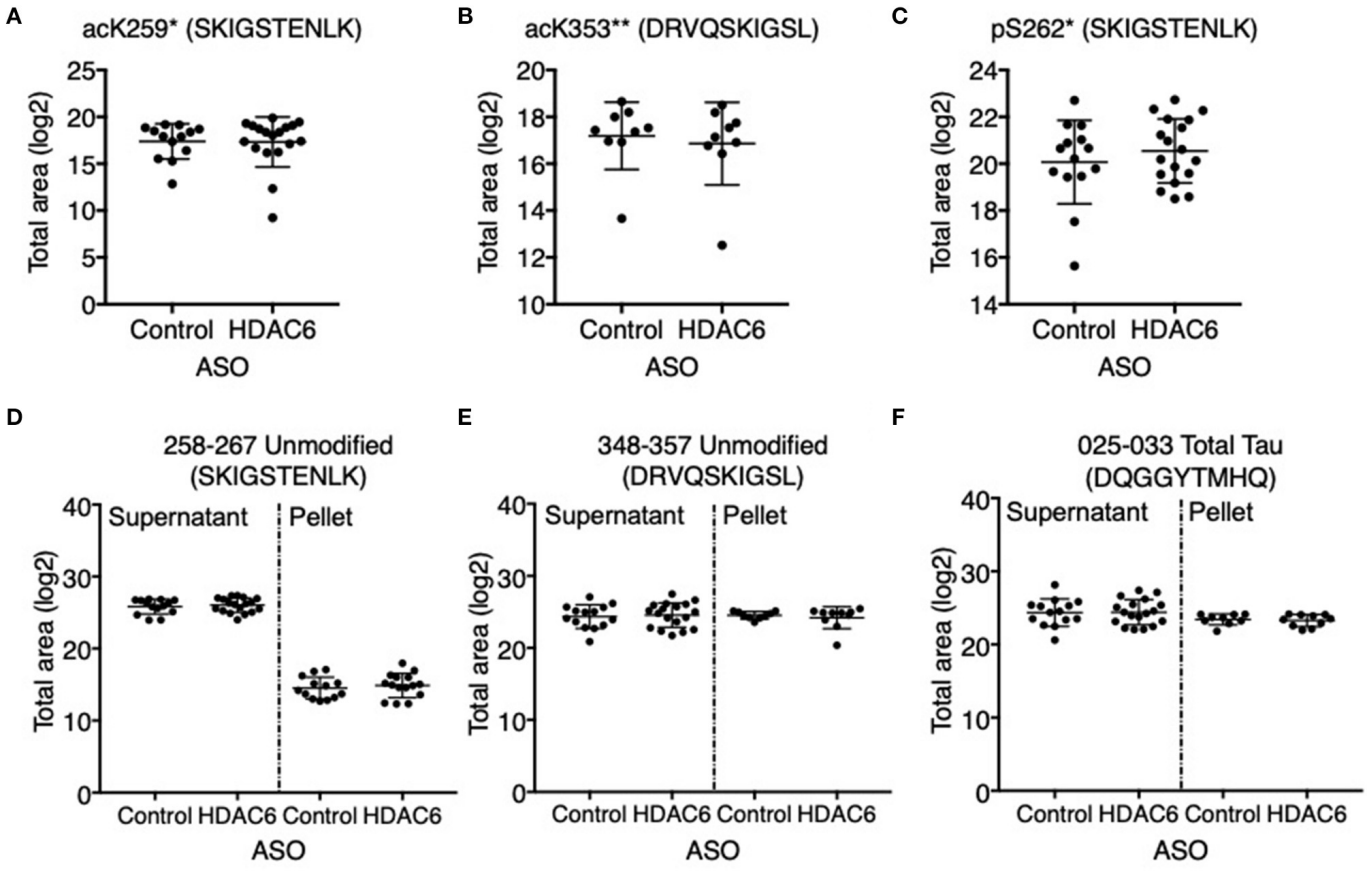
HDAC6 knockdown did not alter acetylation of tau at K259, K353 or phosphorylation at S262 in PS19 mice. Mass spectrometry-based quantitation of **(A)** acK259 tau by peptide SacKIGSTENLK, **(B)** acK353 tau by peptide DRVQSacKIGLS, **(C)** pS262 tau by peptide SKIGpSTENLK, **(D,E)** unmodified KIGS-containing peptides, SKIGSTENLK and DRVQSKIGLS, and **(F)** total tau by a representative peptide, DQGGYTMHQ, in Control ASO- (*n* = 14) and HDAC6 ASO-treated (*n* = 18) PS19 mice. Tau peptide containing pS356 (DRVQSKIGLpS) was not detected and no significant changes were observed for the rest of tau peptides (Supplementary Table 2). *The peptide was only detected in the supernatant of crude homogenate (CH). **The peptide was only detected in the pellet of CH, and only in nine control ASO- and nine HDAC6 ASO-treated samples. The notions of “ac” or “p” in front of K or S, and in peptide sequences indicate the acetylation or phosphorylation sites. Data is presented as mean ± SD and was analyzed using an unpaired non-parametric *t*-test.

We next assessed if HDAC6 KD can increase the acetylation of tau at the KIGS motifs. Western blot analysis of brain lysates with the acetyl-tau specific antibody anti-ac-KIGS did not detect a change in acetyl-tau levels in HDAC6 ASO treated PS19 mice compared to controls (Figures 4A,D; *p* = 0.7644, Supplementary Figure 4). We then tested if phosphorylation at serine residues within the KIGS motifs was altered upon HDAC6 ASO treatment. When probing for tau phosphorylation at S262 (Figures 4C,D, *p* = 0.9905, Supplementary Figure 4) and S324 (Figures 4B,D, *p* = 0.5739, Supplementary Figure 4) we did not detect a change in tau phosphorylation in response to HDAC6 KD. To further confirm the findings, a targeted liquid chromatography-mass spectrometry (LC-MS) assay for simultaneously quantifying either phosphorylated, acetylated or unmodified KIGS-containing tau peptides was developed and used to analyze immunoprecipitation enriched tau protein (36) from brain extracts of PS19 mice treated with HDAC6 or control ASO. Total tau was also measured via inclusion of additional peptides from unmodified tau sequence regions in the assay (Supplementary Table 2). Consistent with the Western blot results, the LC-MS analysis (Figure 5) showed no change in the levels of tau acetylation at K259 (Figure 5A; *p* = 0.9391) and K353 (Figure 5B; *p* = 0.6668) and no change in phosphorylation levels at S262 (Figure 5C; *p* = 0.3972) was seen. Tau peptides phosphorylated at S356 were not detected. There were no changes in unmodified KIGS-containing tau peptides or in total tau (Figures 5D–F). Furthermore, no correlation between acetylation at K259 and phosphorylation at S262 was observed in mass spectrometry-based analysis (data not shown). Together, these data suggest that HDAC6 does not regulate acetylation and phosphorylation at the KIGS motifs of tau *in vivo*.

### Knockdown of HDAC6 Does Not Alter Tau Pathology in Tau Transgenic Mice

**Figure 6:**
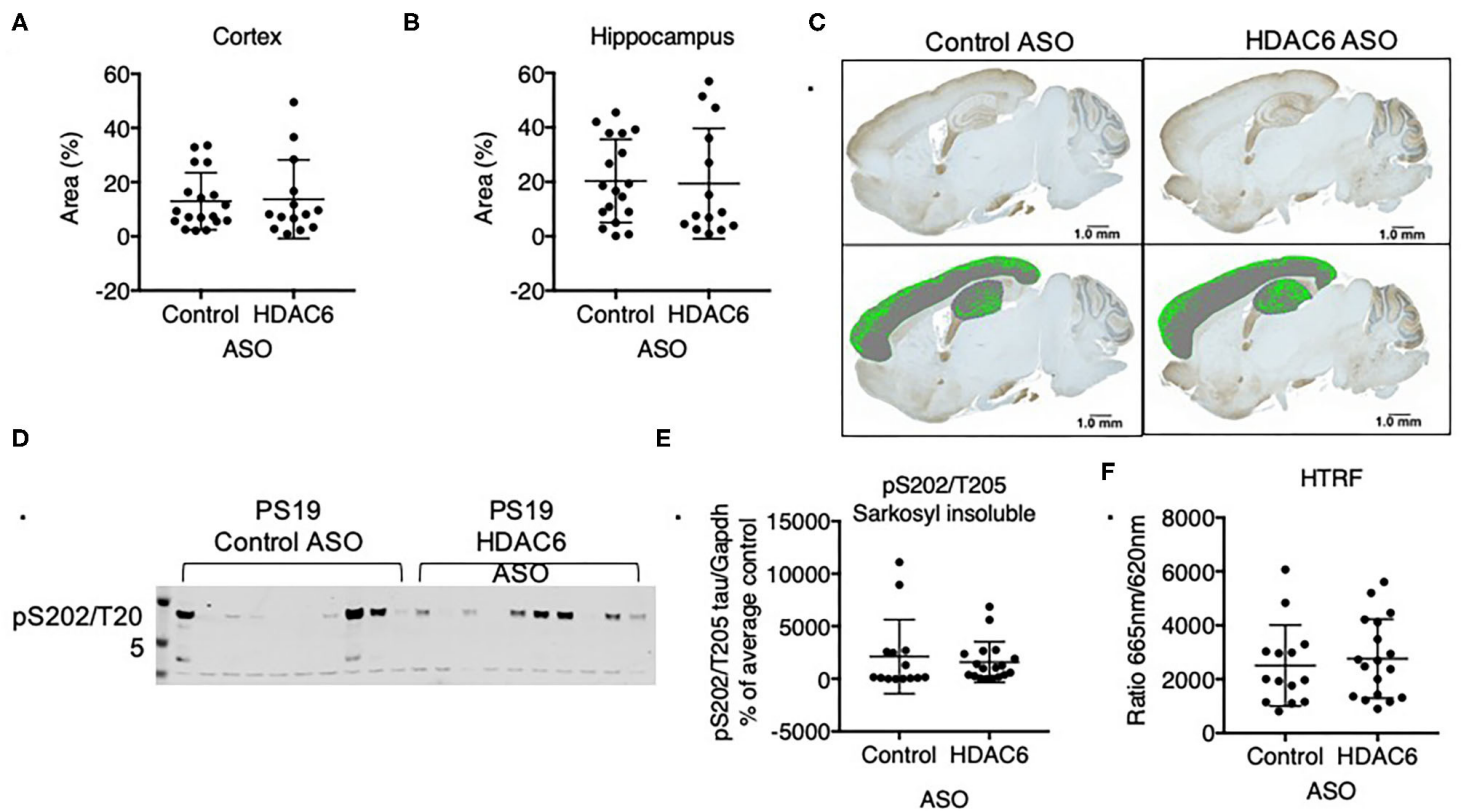
HDAC6 knockdown did not alter levels of pathological tau in PS19 mice. Immunohistochemistry quantification of pS202/T205 tau in **(A)** cortical and **(B)** hippocampal sagittal brain slices of PS19 mice treated with Control or HDAC6 ASO. **(C)** Representative brain slice images (pS202/T205 staining) of PS19 mice treated with Control or HDAC6 ASO. **(D)** Representative Western Blot and **(E)** Western Blot quantification of pS202/T205-tau in PS19 mice treated with Control or HDAC6 ASO. **(F)** HTRF analysis of tau aggregation in brains of PS19 mice treated with Control ASO or HDAC6 ASO. Data is presented as mean ± SD and was analyzed using an unpaired non-parametric *t*-test.

Previous data suggested that increasing acetylation at lysine residues in the KXGS motifs of tau via inhibition of HDAC6 attenuates tau aggregation *in vitro* (11). To test if tau pathology is altered in response to decreased HDAC6 levels we analyzed brains of PS19 mice that were treated for 12 weeks with either HDAC6 ASO or control ASO by immunohistochemistry with an antibody specific for tau phosphorylated at S202 and T205. We detected variable, but significant tau pathology in the cortex and hippocampus (Figure 6C) of mice in both treatment groups. Tau pathology was observed in brain regions expected for this model, however, there was no statistically significant difference in the extent of tau pathology in HDAC6 ASO treated mice as compared to control ASO treated mice (Figures 6A,B; cortex: *p* = 0.8664; hippocampus: *p* = 0.8790, Figure 6C).

We corroborated the lack of treatment effect of HDAC6 ASO on tau pathology by measuring the amount of sarkosyl insoluble, hyperphosphorylated tau via Western blot (Figures 6D,E, Supplementary Figure 6) with a pS202/T205 specific antibody. Consistent with the immunohistochemistry findings insoluble tau levels varied within treatment groups, but there was no difference in the level of insoluble, hyperphosphorylated tau between treatment groups (Figure 6E; *p* = 0.5960). Lastly, we quantified the amount of aggregated tau in the total sarkosyl fraction of brains from PS19 animals treated with HDAC6 or control ASO using a commercially available HTRF-based assay, which employs an anti-tau monoclonal antibody labeled with either terbium-cryptate or d2 (Figure 6F). The specific HTRF signal generated by this antibody combination is proportional to the amount of tau aggregates. Consistent with the Western blot and immunohistochemistry findings, there was no treatment effect of HDAC6 KD on the level of tau aggregates (Figure 6F; *p* = 0.6312). The amount of pathological tau as measured by the HTRF-based immunoassay correlated well with the level of sarkosyl insoluble, hyperphosphorylated tau as measured by Western blot (Supplementary Figure 7).

## Discussion

Using an independent cohort of brains from subjects diagnosed with Alzheimer's disease, we have confirmed that tau acetylation is decreased on KXGS motifs whereas tau phosphorylation is increased as compared to healthy control brains, suggesting a possible inverse interrelationship between these two posttranslational modifications. Furthermore, previous studies suggested that tau acetylation, at least in part, is regulated by HDAC6 activity and that tau acetylation at specific lysine residues can reduce tau aggregation by reducing tau phosphorylation at the nearby serine residues (11). We hypothesized that increasing tau acetylation, by means of reducing levels of HDAC6, would decrease tau phosphorylation and, in turn, the formation of tau aggregates. To test this, we used ASO to reduce HDAC6 expression in brains of PS19 mice, which develop age-dependent tau pathology with significant levels of insoluble filamentous and hyperphosphorylated tau by 6 months of age (28).

We were able to achieve a robust KD of endogenous HDAC6 (~80% reduction of protein levels) with an ASO targeting mouse *Hdac6* mRNA in wild type and PS19 mice. The knockdown of HDAC6 was accompanied by the increased acetylation of two known targets of HDAC6, alpha-tubulin and cortactin, thus demonstrating HDAC6 KD causes a pharmacodynamic response. However, HDAC6 KD did not result in changes in tau acetylation or phosphorylation. Additionally, we did not observe any changes in tau pathology in either the sarkosyl-soluble or -insoluble brain fractions upon HDAC6 KD. Our findings suggest that HDAC6 does not regulate acetylation of tau on KXGS motifs *in vivo* and that reducing levels of HDAC6 does not impact tau pathology in this mouse model.

Our results with HDAC6 ASO are inconsistent with previously published studies performed with HDAC6 small molecule inhibitors that showed a reduction of pathological tau in mouse models of tau pathology (16, 20). This could be due to several factors. ASO are highly specific for the target whereas small molecule inhibitors can be confounded by limited selectivity and potential off-target activity (45, 46). Thus, any effect observed with small molecule HDAC6 inhibitors cannot be conclusively ascribed to the inhibition of HDAC6 activity itself. Additionally, although substantial HDAC6 KD was achieved with the ASO, ~20% expression of HDAC6 protein remained, which might provide enough residual enzymatic activity to deacetylase substrates. On the other hand, small molecule HDAC6 inhibitors, administered at an appropriate dose level and frequency may achieve complete target inhibition.

Furthermore, HDAC6 engages with many cellular substrates including Hsp90, cortactin, tubulin, Ku70, Prx (22, 24, 25, 36, 47) and modulates multiple cellular pathways involved in cellular function. It is thus feasible that disease modifying effects of HDAC6 inhibition are mediated by molecular mechanisms independent of post-translationally altered tau. Lastly, the apparent efficacy observed with small molecule pharmacological approaches targeting HDAC6 could also be due to different treatment duration and the use of different animal models. Although studies utilizing HDAC6 small molecule inhibitors have used similar treatment durations as in our study, they utilized different tau mouse models and different timepoints of treatment initiation relative to the onset of tau pathology. MPT0G211 treatment was started before the onset of tau pathology in 6-month-old 3 × Tg mice and continued for 3 months (17) and Tubastatin-A treatment was initiated in 5-month-old rTg4510 mice with established tau pathology for 2 months (16).

Our data demonstrate that HDAC6 is not involved in deacetylation of human tau on KXGS motifs *in vivo*. This finding is partially supported by other published data. In a recent study, pharmacological inhibition of HDAC6 with ACY-738 was shown to reverse cognitive and synaptic integrity deficits associated with cisplatin treatment (48). HDAC6 inhibition also reversed increases in tau phosphorylation at S202/T205 in cisplatin treated animals (48), but the authors reported no change in tau acetylation at K280 upon HDAC6 inhibition, similar to the unaltered acetylation levels at K259 and K353 observed in our study. It cannot be ruled out that HDAC6 is involved in the deacetylation of other lysine residues on tau which could influence tau phosphorylation at adjacent sites, and it should be noted that we have not studied lysine residues outside of KXGS motifs. In other studies, in which HDAC6 inhibitors were shown to be efficacious in mouse models of tau pathology, the status of tau acetylation has not been reported (16, 20) which makes it difficult to establish a correlation between the acetylation and the phosphorylation of tau.

Other reports suggest that HDAC6's involvement in acetylation of tau can be dependent on the cellular environment. In normal mouse primary cortical neurons tau is not highly acetylated on K280. However, when challenged with neuroinflammatory factors endogenous tau mislocalized to neuritic foci, also known as neuritic beads, and was hypophosphorylated and hyperacetylated on K280. In this context, inhibition or genetic deletion of HDAC6 resulted in a small increase in tau acetylation as well as suppression of the neuritic bead formation (49). Although the neuroinflammatory environment changes in brains of PS19 mice with age (50) the acetylation status of tau at K280 does not (51), indicating that changes in the acetylation of tau induced by neuroinflammatory factors *in vitro* are not recapitulated *in vivo*. This also suggests that the neuroprotective action of HDAC6 inhibition *in vitro* may not be effective *in vivo*.

Taken together, our data demonstrate that the regulation of tau acetylation, at least on lysines 259 and 353, is independent of HDAC6 activity *in vivo* and that selective reduction of HDAC6 activity does not impact tau pathology in PS19 mice.

## Data Availability Statement

The original contributions presented in the study are included in the article/Supplementary Material, further inquiries can be directed to the corresponding author/s.

## Ethics Statement

The animal study was reviewed and approved by Biogen IACUC; guidelines and regulations (Cambridge Ordinance 1086, PHS Policy, AWA/AWAR, and the Guide).

## Author Contributions

AV: investigation and formal analysis. VB and BB: investigation, formal analysis, visualization, and writing—review & editing. GM: supervision, investigation, formal analysis, and visualization. SH: investigation, formal analysis, and visualization. RW and FR: supervision and writing—review & editing. KL: investigation, formal analysis, and writing—review & editing. HA: supervision. OG: writing—review & editing. HH: conceptualization, supervision, and writing—original draft. All authors contributed to the article and approved the submitted version.

## Conflict of Interest

VB, BB, GM, SH, RW, HA, OG, and HH are employed by Biogen. AV and OG were also employed by Biogen at the time of the study. KL and FR are employed by Ionis Pharmaceuticals.

## Supplementary Material

Supplementary Figure 1 Full images of blots presented in Figure 1.

Supplementary Figure 2 Full images of blots presented in Figure 2.

Supplementary Figure 3 Full images of blots presented in Figure 3.

Supplementary Figure 4 Full images of blots presented in Figure 4.

Supplementary Figure 5 **(A)** Western Blot analysis of acetyl-cortactin and GADPH in HDAC6 KO (*n* = 4) and WT (*n* = 2) mouse hemi-brain. **(B,C)** Western Blot analysis of cortactin and acetyl-cortactin in HDAC6 Heterozygous (*n* = 3), Hemizygous (*n* = 2), and WT (*n* = 2) mouse hemibrains following immunoprecipitation with either Santa Cruz or Millipore cortactin antibody. Arrows in **(B,C)** point to the ~50 kDa cortactin band (see Methods section). **(D,E)** Full images of blots presented in **(B,C)**.

Supplementary Figure 6 Full images of blots presented in Figure 6.

Supplementary Figure 7 Correlation between Western Blot and HTRF measures of tau aggregation. Data is presented as a linear regression. *n* = 14 and *n* = 18 for Control ASO- and HDAC6 ASO-treated PS19 mice, respectively.

Supplementary Figure 8 *In-situ* hybridization analysis of HDAC6 mRNA expression using RNAScope method. Representative images of cortex are shown. **(A,C)** Hybridization of the HDAC6-specific probe is visualized as brown dots; cell nuclei are blue (hematoxylin nuclear counterstain). **(B,D)** Image analysis mask corresponding to images **(A,C)**, respectively, and showing the detection of the probe hybridization in yellow. **(A)** Control ASO-treated animal and **(B)** corresponding image analysis mask; **(C)** HDAC6 ASO-treated animal and **(D)** corresponding image analysis mask.

Supplementary Table 1 Information for antibodies used in the present study. Antibody name, source, product number, and dilution (presented as a ratio of 1:X) are listed.

Supplementary Table 2 Peptides assayed by the targeted LC-MS/PRM method. KIGS motifs, peptide sequences, amino acid positions in tau protein, and PTM description are listed.
