# Supplementary figures and images for: Antisense Oligonucleotide-Mediated Reduction of HDAC6 Does Not Reduce Tau Pathology in P301S Tau Transgenic Mice
Source: Front Neurol. 2021 Jun 28;12:624051. doi: 10.3389/fneur.2021.624051 (PMC8273312; doi:10.3389/fneur.2021.624051)

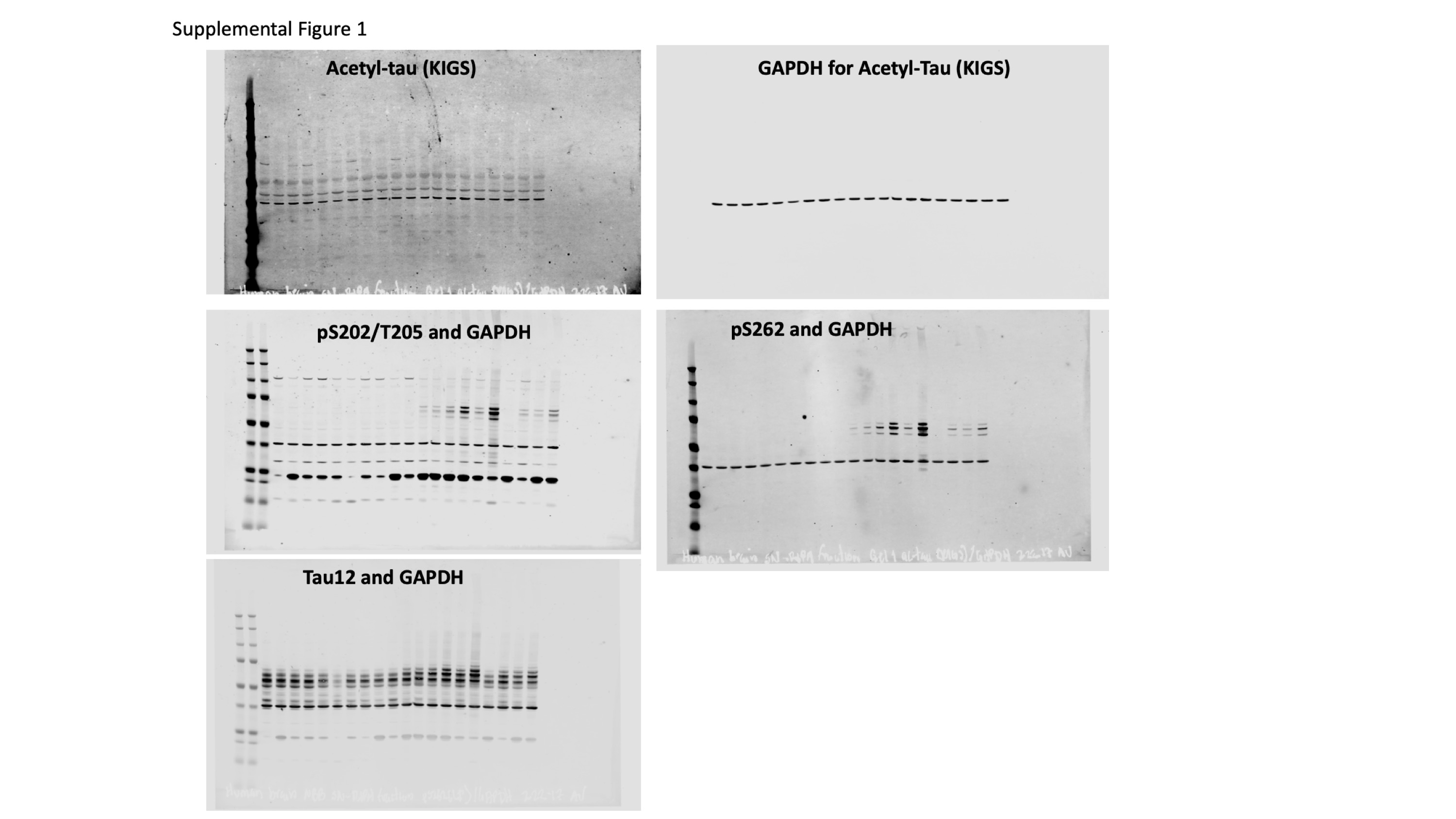

Supplement: Supplementary Figure 1 — Full images of blots presented in Figure 1. [file Image_1.JPEG]

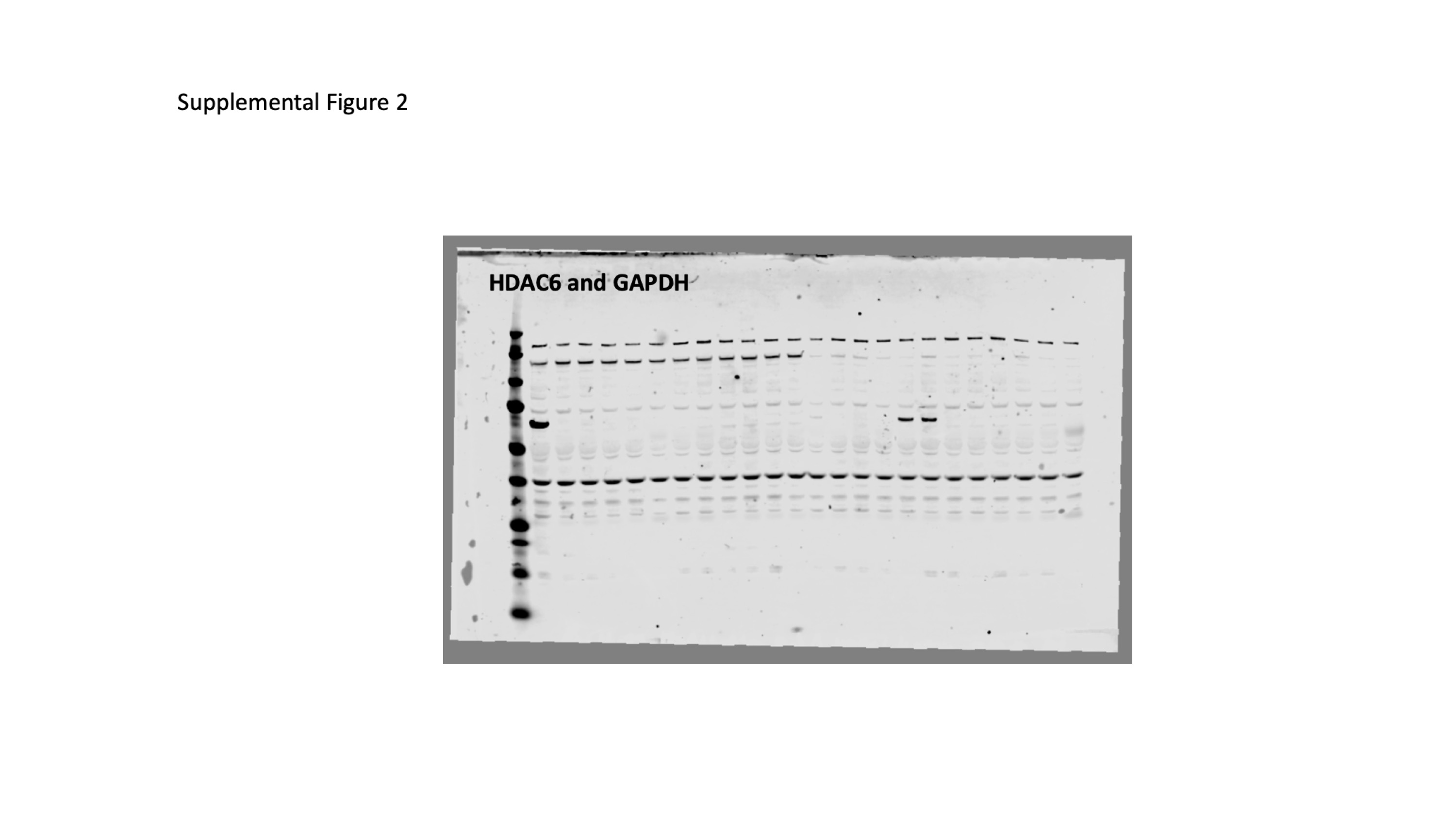

Supplement: Supplementary Figure 2 — Full images of blots presented in Figure 2. [file Image_2.JPEG]

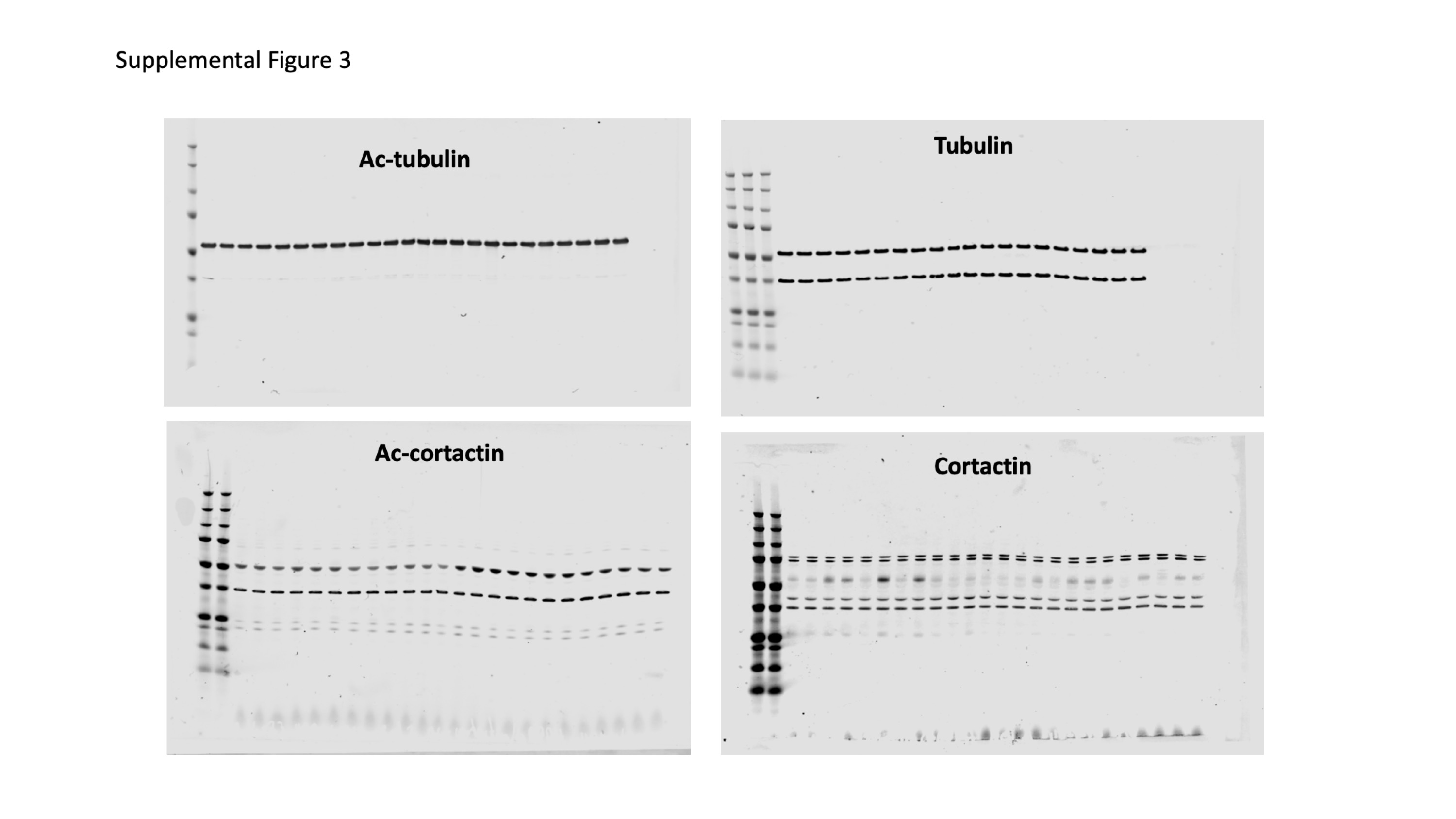

Supplement: Supplementary Figure 3 — Full images of blots presented in Figure 3. [file Image_3.JPEG]

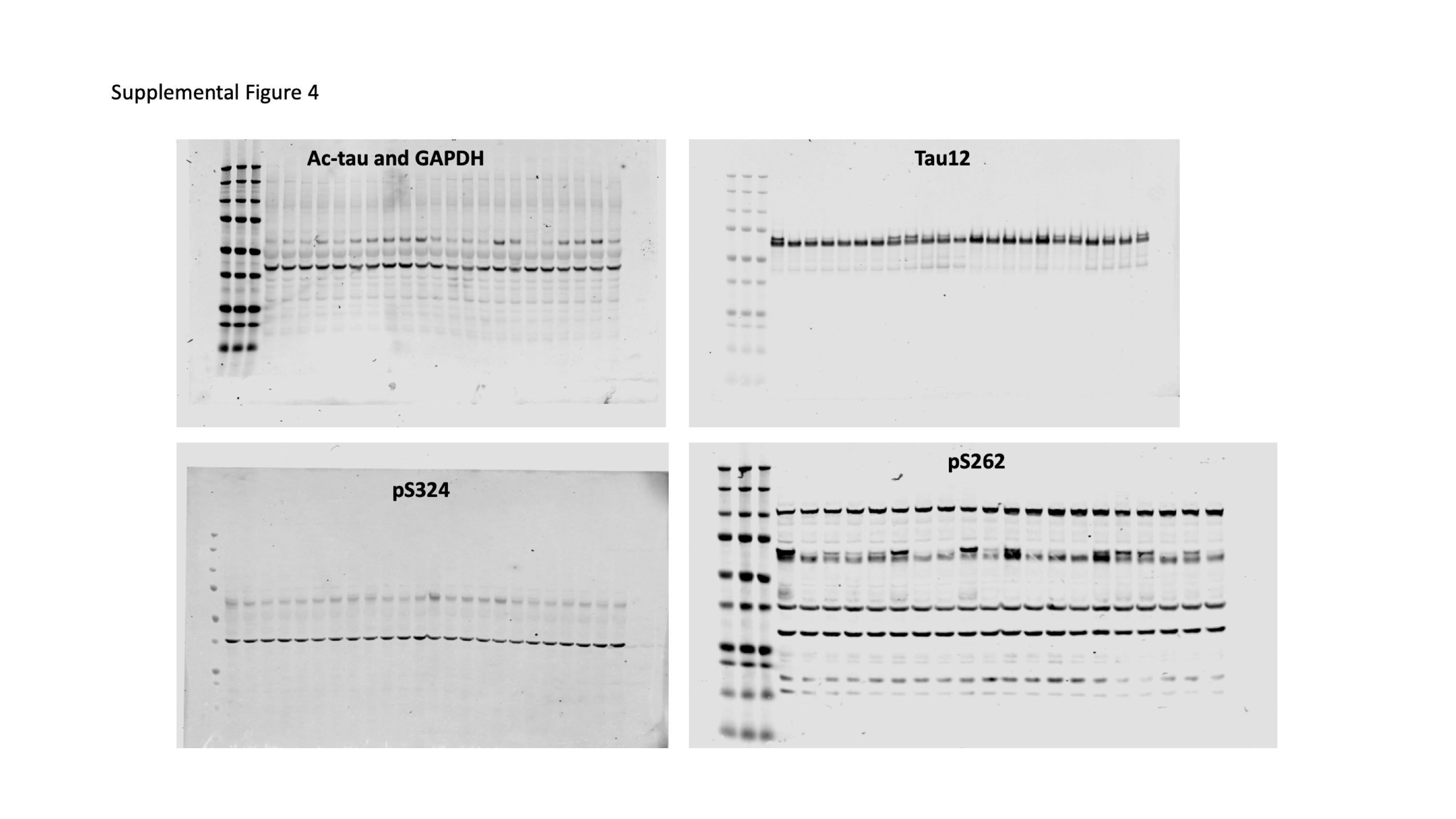

Supplement: Supplementary Figure 4 — Full images of blots presented in Figure 4. [file Image_4.JPEG]

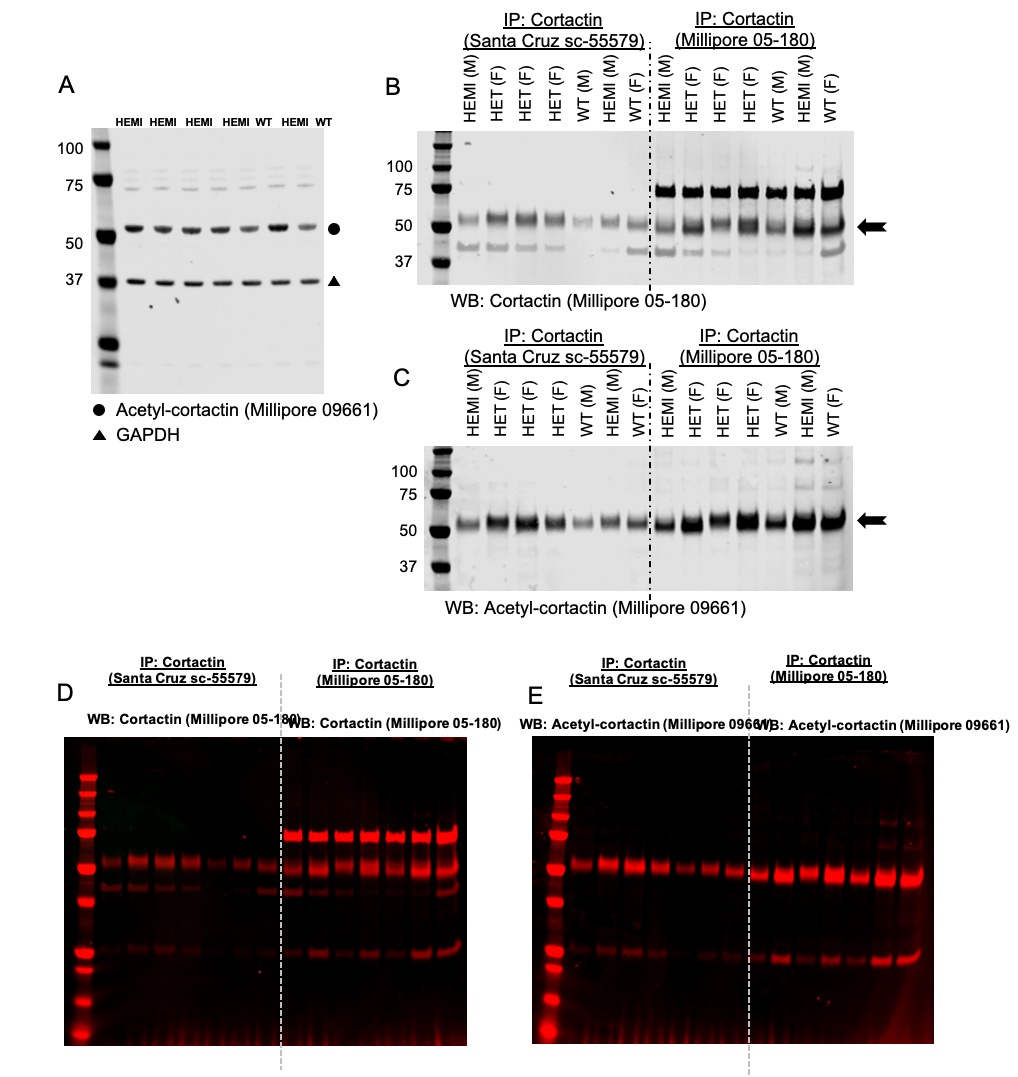

Supplement: Supplementary Figure 5 — (A) Western Blot analysis of acetyl-cortactin and GADPH in HDAC6 KO (n = 4) and WT (n = 2) mouse hemi-brain. (B,C) Western Blot analysis of cortactin and acetyl-cortactin in HDAC6 Heterozygous (n = 3), Hemizygous (n = 2), and WT (n = 2) mouse hemibrains following immunoprecipitation with either Santa Cruz or Millipore cortactin antibody. Arrows in (B,C) point to the ~50 kDa cortactin band (see Methods section). (D,E) Full images of blots presented in (B,C). [file Image_5.jpg]

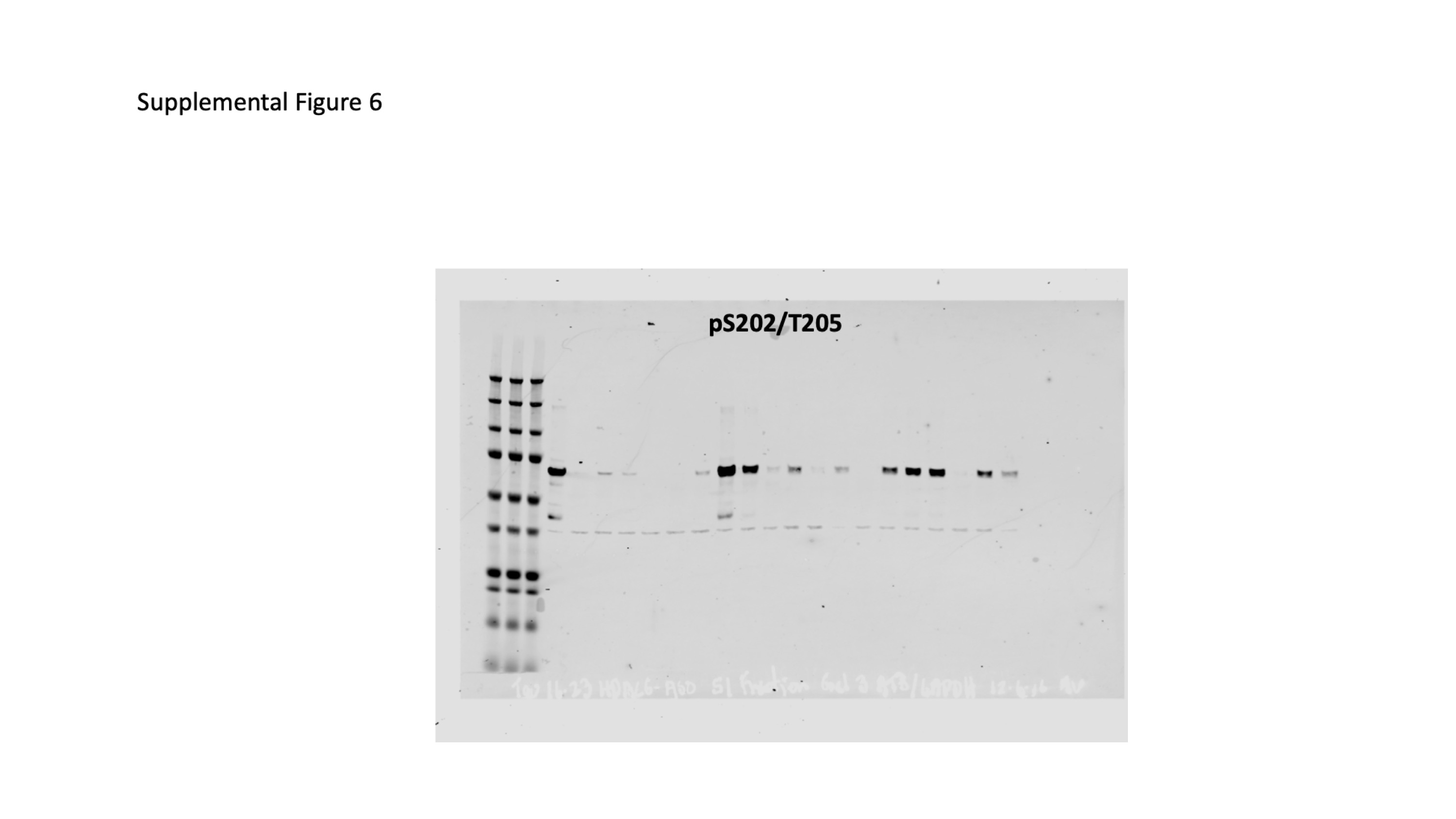

Supplement: Supplementary Figure 6 — Full images of blots presented in Figure 6. [file Image_6.jpeg]

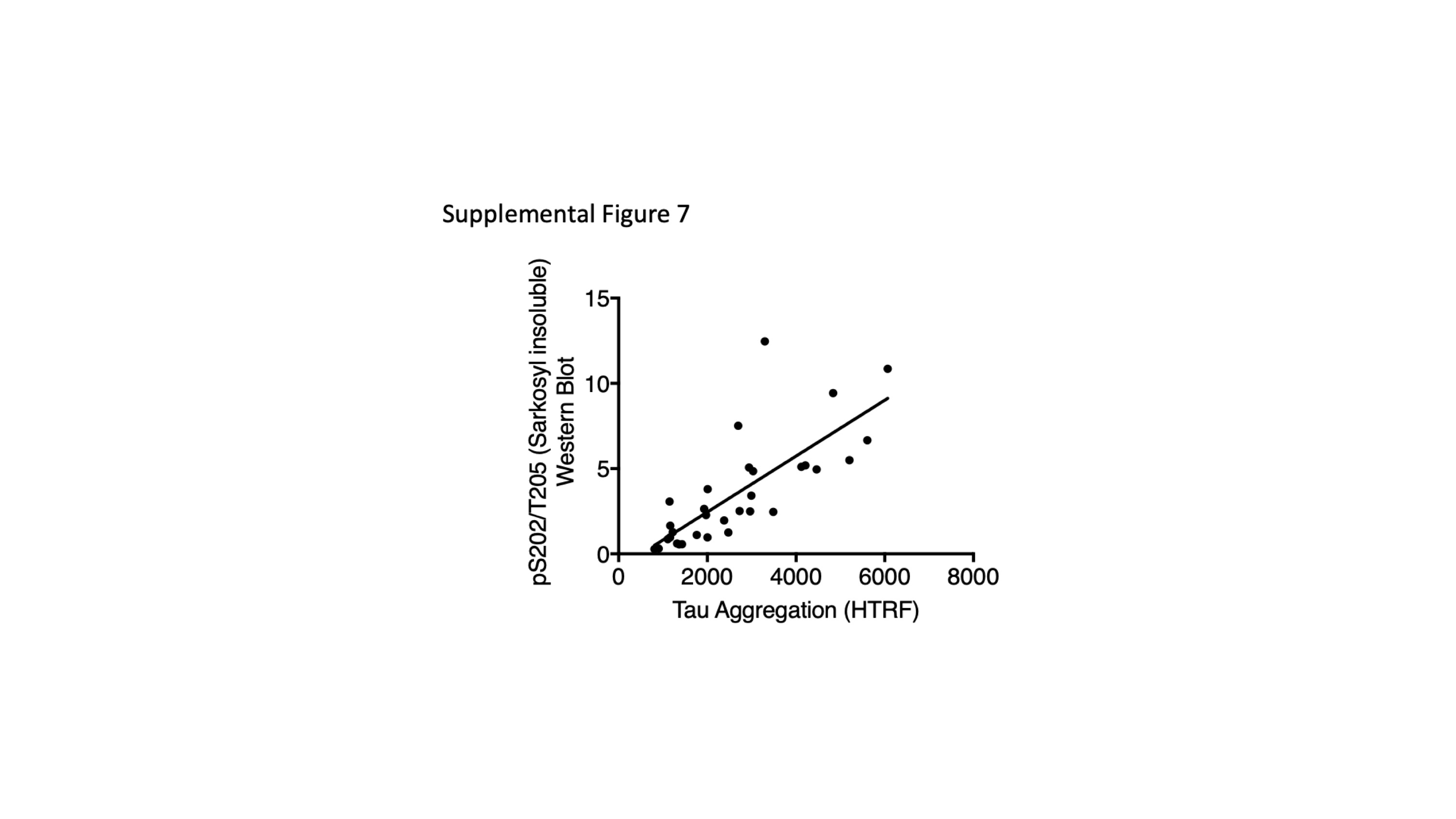

Supplement: Supplementary Figure 7 — Correlation between Western Blot and HTRF measures of tau aggregation. Data is presented as a linear regression. n = 14 and n = 18 for Control ASO- and HDAC6 ASO-treated PS19 mice, respectively. [file Image_7.jpeg]

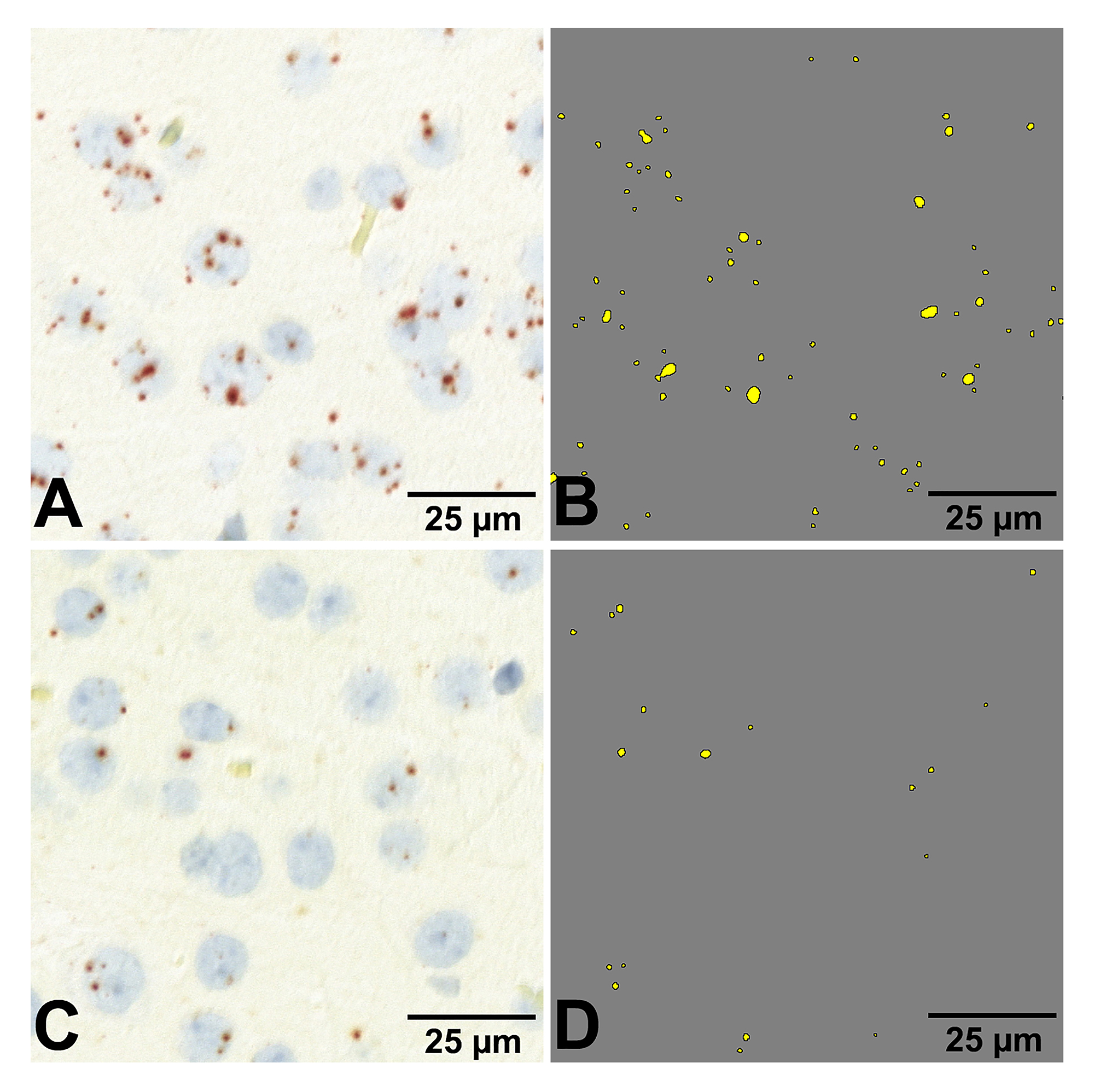

Supplement: Supplementary Figure 8 — In-situ hybridization analysis of HDAC6 mRNA expression using RNAScope method. Representative images of cortex are shown. (A,C) Hybridization of the HDAC6-specific probe is visualized as brown dots; cell nuclei are blue (hematoxylin nuclear counterstain). (B,D) Image analysis mask corresponding to images (A,C), respectively, and showing the detection of the probe hybridization in yellow. (A) Control ASO-treated animal and (B) corresponding image analysis mask; (C) HDAC6 ASO-treated animal and (D) corresponding image analysis mask. [file Image_8.jpeg]
