# Supplementary material for: Antisense Oligonucleotide-Mediated Reduction of HDAC6 Does Not Reduce Tau Pathology in P301S Tau Transgenic Mice
Source: Front Neurol. 2021 Jun 28;12:624051. doi: 10.3389/fneur.2021.624051 (PMC8273312; doi:10.3389/fneur.2021.624051)
Supplement: Supplementary Table 1 — Information for antibodies used in the present study. Antibody name, source, product number, and dilution (presented as a ratio of 1:X) are listed. [file Table_1.docx]

**Supplemental Table 1**

| **Antibody**​ | **Source**​ | **Product Number**​ | **Dilution 1:**​ |
| --- | --- | --- | --- |
| anti-tau cloneTau12​ | Millipore​ | 2241​ | 1000​ |
| anti-pS202/T205 tau AT8​ | Life Technologies​ | MN1020​ | 1000​ |
| anti-pS262 tau​ | Life Technologies​ | 44-750G​ | 1000​ |
| anti-pS324 tau ​ | abcam​ | 109401​ | 1000​ |
| anti-pS356 tau​ | Life Technologies​ | 44-751G​ | 1000​ |
| anti-acetylated tau KIGS​ | The Mayo Clinic​ | gift from L. Petrucelli​ | 1000​ |
| anti-HDAC6​ | Millipore​ | 07-732​ | 1000​ |
| anti-cortactin (p80/p85)​ | Millipore​ | 05-180​ | 1000​ |
| anti-cortactin (H-5)​ | Santa Cruz​ | sc-55579​ | 1000​ |
| anti-acetyl-cortactin​ | Millipore​ | 09881​ | 1000​ |
| anti-a-tubulin ​ | Cell Signaling​ | 11H10​ | 10000​ |
| anti-acetyl-a-tubulin ​ | abcam​ | 246110​ | 10000​ |
| anti-GAPDH (6C5)​ | abcam​ | 8245​ | 10000​ |
| anti-GAPDH (EPR16891)​ | abcam​ | 181602​ | 10000​ |
