# Supplementary material for: Antisense Oligonucleotide-Mediated Reduction of HDAC6 Does Not Reduce Tau Pathology in P301S Tau Transgenic Mice
Source: Front Neurol. 2021 Jun 28;12:624051. doi: 10.3389/fneur.2021.624051 (PMC8273312; doi:10.3389/fneur.2021.624051)
Supplement: Supplementary Table 2 — Peptides assayed by the targeted LC-MS/PRM method. KIGS motifs, peptide sequences, amino acid positions in tau protein, and PTM description are listed. [file Table_2.docx]

**Supplemental Table 2**

| **tau Domain**​ | **Peptide Sequence**​ | **Position Tau-F**​ | **Comment**​ |
| --- | --- | --- | --- |
| KIGS (259-262)​ | SKIGSTENLK​ | 258 - 267​ | Unmodified​ |
|  | Sac**K**IGSTENLK​ | 258 - 267​ | Acetylated at K259​ |
|  | SKIG**pS**TENLK​ | 258 - 267​ | Phosphorylated at S262​ |
|  | IGSTENLK​ | 260 - 267​ | Unmodified​ |
|  | IG**pS**TENLK​ | 260 - 267​ | Phosphorylated at S262​ |
| KIGS (353-362)​ | DRVQSKIGSL​ | 348 - 357​ | Unmodified​ |
|  | DRVQS**acK**IGSL​ | 348 - 357​ | Acetylated at K353​ |
|  | DRVQSKIG**pS**L​ | 348 - 357​ | Phosphorylated at S356​ |
| Total tau​ | DQGGYTMHQ​ | 25-33​ | Unmodified​ |
|  | LQTAPVPoxMPDLK​ | 185-196​ | Oxidized at M192​ |
|  | TDHGAEIVYK​ | 297-306​ | Unmodified​ |
